# Supplementary figures and images for: Skeletal muscle-kidney crosstalk in a cohort of critical illness survivors
Source: PLoS One. 2026 Jan 16;21(1):e0339795. doi: 10.1371/journal.pone.0339795 (PMC12810808; doi:10.1371/journal.pone.0339795)

**Supporting Information**

**
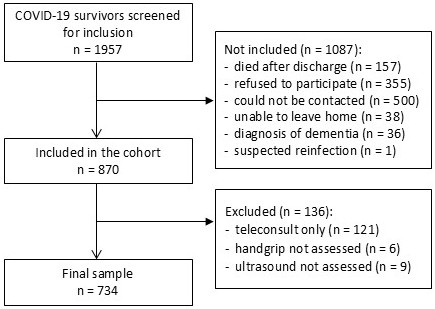
**

**S1 Figure**. Flowchart.

Supplement: S1 Fig — (DOCX) [file pone.0339795.s001.docx]
