# Supplementary material for: Skeletal muscle-kidney crosstalk in a cohort of critical illness survivors
Source: PLoS One. 2026 Jan 16;21(1):e0339795. doi: 10.1371/journal.pone.0339795 (PMC12810808; doi:10.1371/journal.pone.0339795)
Supplement: S2 Table — eGFR. estimated glomerular filtration rate. (DOCX) [file pone.0339795.s002.docx]

**Supporting Information**

**S2 Table**. Missing data.

|  | **Absolute (n)** | **Relative (%)** |
| --- | --- | --- |
| **Clinical** |  |  |
| Age | 0 | 0.0 |
| Sex | 0 | 0.0 |
| Ethnicity | 5 | 0.7 |
| Body mass index | 53 | 7.1 |
| **Skeletal muscle** |  |  |
| Handgrip strength (kg) | 0 | 0.0 |
| Calf circumference (cm) | 3 | 0.4 |
| Gait speed (m/s) | 38 | 5.1 |
| Quadriceps ultrasound (mm) | 0 | 0.0 |
| **Kidney function** |  |  |
| eGFR (ml/min/1.73 m^2^) | 0 | 0.0 |
| Albuminuria | 147 | 20.5 |
| Creatinine | 26 | 3.5 |
| Urinary sediment | 26 | 3.5 |

eGFR**.** estimated glomerular filtration rate.
