# Supplementary material for: Skeletal muscle-kidney crosstalk in a cohort of critical illness survivors
Source: PLoS One. 2026 Jan 16;21(1):e0339795. doi: 10.1371/journal.pone.0339795 (PMC12810808; doi:10.1371/journal.pone.0339795)
Supplement: S3 Table — CI, confidence interval; eGFR, estimated glomerular filtration rate; UACR, urinary albumin-to-creatinine ratio. Multiple linear regression analysis adjusted for age (continuous), sex, and diabetes. We defined younger survivors as those <60 years. Variables considered for the sensitivity analyses were not included in the adjusted model. (DOCX) [file pone.0339795.s003.docx]

**Supporting Information**

**S3 Table**. Sensitivity analysis for the association between skeletal muscle parameters and kidney function markers.

| **Skeletal muscle** | **Kidney function** | | | |
| --- | --- | --- | --- | --- |
|  | eGFR (mL/min/1.73m^2^) | | UACR (mg/g) | |
|  | **β (95% CI)** | ***p*-value** | **β (95% CI)** | ***p*-value** |
| **Excluding younger survivors** (n = 322) | |  |  |  |
| Handgrip strength (kg) | 0.01 (-0.03 to 0.04) | 0.658 | 0.05 (-0.02 to 0.11) | 0.158 |
| Calf circumference (cm) | 0.09 (-0.44 to 0.62) | 0.727 | -0.23 (-1.44 to 0.97) | 0.704 |
| Gait speed (sec) | -0.20 (-0.73 to 0.34) | 0.475 | -0.84 (-12.62 to 10.95) | 0.889 |
| Rectus femoris (mm) | 0.38 (-0.33 to 1.08) | 0.292 | -0.73 (-2.28 to 0.82) | 0.357 |
| Vastus intermedius (mm) | 0.57 (-0.07 to 1.21) | 0.082 | -0.01 (-1.36 to 1.34) | 0.989 |
| **Excluding male survivors** (n = 343) | |  |  |  |
| Handgrip strength (kg) | 0.02 (-0.01 to 0.05) | 0.161 | -0.03 (-0.08 to 0.03) | 0.304 |
| Calf circumference (cm) | 0.24 (-0.23 to 0.70) | 0.311 | 0.31 (-0.71 to 1.32) | 0.550 |
| Gait speed (sec) | 0.18 (-0.43 to 0.79) | 0.561 | 1.33 (0.14 to 2.52) | 0.028 |
| Rectus femoris (mm) | 0.14 (-0.55 to 0.82) | 0.692 | 0.75 (-0.75 to 2.25) | 0.328 |
| Vastus intermedius (mm) | 0.53 (-0.12 to 1.17) | 0.109 | 0.89 (-0.45 to 2.22) | 0.192 |

CI, confidence interval; eGFR, estimated glomerular filtration rate; UACR, urinary albumin-to-creatinine ratio.

Multiple linear regression analysis adjusted for age (continuous), sex, and diabetes.

We defined younger survivors as those <60 years.

Variables considered for the sensitivity analyses were not included in the adjusted model.
