# Supplementary material for: Skeletal muscle-kidney crosstalk in a cohort of critical illness survivors
Source: PLoS One. 2026 Jan 16;21(1):e0339795. doi: 10.1371/journal.pone.0339795 (PMC12810808; doi:10.1371/journal.pone.0339795)
Supplement: Attachment — Submitted filename: Response letter.pdf [file pone.0339795.s008.pdf]

São Paulo, December 2025

To: Dr. Diego Moriconi
Associate Editor
PLOS ONE
Re: PONE-D-25-13841

Dear Editor,

Thank you very much for allowing us to submit a revised version of our manuscript "**Skeletal Muscle-Kidney Crosstalk in a Cohort of Critical Illness Survivors**". On behalf of all authors, I would like to thank the editor and the reviewers for the careful analysis of our manuscript and their thoughtful insights. All comments and suggestions have been carefully addressed, and the manuscript was revised and amended accordingly. Please, find below a detailed list of specific responses to each of the points raised by the reviewers. The changes made in the text are highlighted in red so that the revisions can be easily identified.

We are looking forward to hearing from you regarding the status of our revision, which we hope will reach the standards for publication of this esteemed journal. If you have any other questions regarding our manuscript, please, do not hesitate to contact me.

Best regards,

Heitor S. Ribeiro, PhD

Laboratório de Investigação (LIM) 12, Serviço de Nefrologia,
Faculdade de Medicina da Universidade de São Paulo,
São Paulo, Brazil, Postal Code: 01246-903
Office Phone: +551130617343
Email: heitorribeiro@usp.br

Reviewer #1

1. Comment: I congratulate the authors on the changes made to the manuscript.

1.1 Response: We sincerely thank the reviewer for their time and thoughtful comments on our manuscript. We hope that this revised version meets a higher standard of quality.

2. Comment: The concluding sentence stating that sarcopenia is associated with a lower eGFR (page 15, lines 320 to 322) is not fully supported by the results, since in the binary logistic regression, after adjusting for age, sex, diabetes, and ICU admission, sarcopenia was not associated with eGFR.

I suggest either tempering the conclusion (the conclusion in the abstract is more appropriate)

2.1 Response: We appreciate the reviewer comment and agree with it. The conclusion has been changed.

2.2 Changes in Conclusions:

> "In survivors of moderate to severe COVID-19 hospitalization, skeletal muscle mass was associated with eGFR, whereas sarcopenia *per se* was not independently associated with poor kidney function. Additionally, several muscle mass parameters were significantly associated with eGFR, but not with UACR. These findings suggest an interaction between skeletal muscle and kidney function."

3. Comment: "Or conducting further analyses to try to find and demonstrate a statistically significant link between muscle and eGFR. :

- Multiple linear or polynomial regression to study the impact of sarcopenia, adjusted for other variables, on eGFR.
- Test whether the association between muscle mass (as measured by ultrasound) and eGFR, found in univariate analysis, persists in multivariate analysis."

3.1 Response: We have previously conducted such analysis adjusted for confounders. Regarding the multivariate analysis, we believe it is not appropriate because there is high collinearity between skeletal muscle mass markers and physical function, instead we adjusted for clinically relevant confounders such as age, sex, and diabetes.
